# Relationship between de novo lipogenesis and serum sex hormone binding globulin in humans

**DOI:** 10.1111/cen.14459

**Published:** 2021-03-28

**Authors:** Pomme I. H. G. Simons, Olivier Valkenburg, Ine Telgenkamp, Koen M. van der Waaij, David M. de Groot, Pandichelvam Veeraiah, Judith A. P. Bons, Marja‐Riitta Taskinen, Jan Borén, Patrick Schrauwen, Joost H. W. Rutten, David Cassiman, Casper G. Schalkwijk, Coen D. A. Stehouwer, Vera B. Schrauwen‐Hinderling, Leanne Hodson, Martijn C. G. J. Brouwers

**Affiliations:** ^1^ Division of Endocrinology and Metabolic Diseases Department of Internal Medicine Maastricht University Medical Centre Maastricht The Netherlands; ^2^ Laboratory for Metabolism and Vascular Medicine Maastricht University Maastricht The Netherlands; ^3^ CARIM School for Cardiovascular Diseases Maastricht University Maastricht The Netherlands; ^4^ Department of Reproductive Medicine Maastricht University Medical Centre Maastricht The Netherlands; ^5^ Department of Nutrition and Movement Sciences Maastricht University Maastricht Netherlands; ^6^ Department of Radiology and Nuclear Medicine Maastricht University Maastricht Netherlands; ^7^ Central Diagnostic Laboratory Maastricht University Medical Centre Maastricht Netherlands; ^8^ Research Program, Unit Clinical and Molecular Metabolism University of Helsinki Helsinki Finland; ^9^ Department of Molecular and Clinical Medicine University of Gothenburg Gothenburg Sweden; ^10^ Department of Internal Medicine Radboud University Medical Centre Nijmegen The Netherlands; ^11^ Department of Gastroenterology‐Hepatology and Metabolic Centre University Hospital Leuven Leuven Belgium; ^12^ Division of General Internal Medicine Department of Internal Medicine Maastricht University Medical Centre Maastricht The Netherlands; ^13^ Oxford Centre for Diabetes, Endocrinology and Metabolism University of Oxford Oxford United Kingdom; ^14^ National Institute for Health Research Oxford Biomedical Research Centre Oxford University Hospitals Foundation Trust Oxford United Kingdom

**Keywords:** de novo lipogenesis, liver fat, non‐alcoholic fatty liver disease, polycystic ovary syndrome, sex hormone binding globulin, stable isotopes

## Abstract

**Objective:**

Obesity and liver fat are associated with decreased levels of serum sex hormone binding globulin (SHBG). Laboratory studies suggest that hepatic de novo lipogenesis (DNL) is involved in the downregulation of SHBG synthesis. The aim of the present study was to address the role of DNL on serum SHBG in humans.

**Design:**

A cross‐sectional study examining the association between DNL, measured by stable isotopes, and serum SHBG, stratified by sex.

**Participants:**

Healthy men (*n* = 34) and women (*n* = 21) were combined from two cross‐sectional studies. Forty‐two per cent of participants had hepatic steatosis, and the majority were overweight (62%) or obese (27%).

**Results:**

DNL was inversely associated with SHBG in women (β: −0.015, 95% CI: −0.030; 0.000), but not in men (β: 0.007, 95% CI: −0.005; 0.019) (*p* for interaction = .068). Adjustment for study population, age and body mass index did not materially change these results, although statistical significance was lost after adjustment for serum insulin.

**Conclusions:**

An inverse association between DNL and SHBG may explain the decreased SHBG levels that are observed in obesity, at least in women.

## 1 INTRODUCTION

Obesity has become a worldwide health burden that is associated with many health concerns including hypertension, dyslipidaemia, type 2 diabetes, non‐alcoholic fatty liver disease, cardiovascular disease, gout, osteoarthritis, fractures and gall bladder disease.^1^, ^2^, ^3^ Individuals with obesity are commonly characterized by low serum sex hormone binding globulin (SHBG) levels.^4^ SHBG is a liver‐specific glycoprotein that binds sex hormones in blood and thereby regulates their bioavailability.^5^ The exact mechanism by which obesity leads to a decrease in serum SHBG levels in humans is not fully understood, although it is likely to be multifactorial. We and others recently showed that a weight reduction programme was associated with an increase in serum SHBG levels.^4^, ^6^ Furthermore, the change in intrahepatic lipid (IHL) content was inversely associated with serum SHBG levels.^4^

Excessive accumulation of IHL in obesity can be explained by an increased conversion of glucose to fat (ie de novo lipogenesis [DNL]) and an increased flux of free fatty acids (FFA) from insulin‐resistant adipose tissue to the liver.^7^ Of interest, previous in vitro studies and mice studies have demonstrated that monosaccharide‐induced DNL reduced serum SHBG levels.^8^ Furthermore, palmitate—a saturated fatty acid that is the principal end product of DNL—directly reduced SHBG expression in HepG2 cells.^8^

The aim of the present study was to extrapolate these experimental data to the human situation. For this, we determined the relationship between DNL, assessed by stable isotopes (the gold standard), and serum SHBG, corrected for potential confounding factors.

## 2 MATERIALS AND METHODS

### 2.1 Study design

In this cross‐sectional study, data from two previously published cohorts in Oxford (UK) and Maastricht (The Netherlands) were combined.^9^, ^10^ Both studies were performed according to the Declaration of Helsinki^11^ and approved by the Medical Ethical Committee of Maastricht University Medical Centre or the Portsmouth Clinical Research Ethics Committee. All participants gave written informed consent prior to participation.

### 2.2 Oxford study population

This study originally aimed to assess the effect of insulin resistance on the synthesis and partitioning of intrahepatic fatty acids. For this purpose, healthy individuals were included when they had a body mass index (BMI) <30 kg/m^2^, did not use medication affecting lipid or glucose metabolism and did not excessively smoke or consume alcohol.^9^ Individuals with high serum triglyceride (TG) levels (>4 mmol/L) were excluded from the present study, as high TG affect the reliability of DNL assessment.^10^, ^12^ All measurements were performed after an overnight fast, and individuals were asked not to consume foods rich in ^13^C or alcohol and to avoid strenuous exercise.^9^

Anthropometrics, measurements of serum lipids, insulin and glucose and quantification of IHL content by proton magnetic resonance spectroscopy (^1^H‐MRS) were done as described previously.^9^ IHL content was expressed as the ratio CH_2_/H_2_0. SHBG in the Oxford study population and the Maastricht study population was measured with an automated chemiluminescent immunometric assay on the Immulite XPi instrument (Siemens Healthcare Diagnostics) in heparinized plasma and serum, respectively (for practical reasons, SHBG is further referred to as ‘serum SHBG’).

DNL was quantified by oral ingestion of deuterated water (${\;}^{2}H_{2}O$) (3 g/kg body water) the evening prior to the measurements and throughout the measurement day.^9^ The incorporation of deuterium from ${\;}^{2}H_{2}O$ in plasma water into very‐low‐density lipoprotein (VLDL)‐TG palmitate is representative of newly synthesized fatty acids from a non‐lipid precursor, and, hence, a marker of DNL. This was measured with gas chromatography‐mass spectrometry (Finnigan GasBench II Thermo Fisher Scientific).^9^

### 2.3 Maastricht study population

This study was primarily conducted to establish a ^1^H‐MRS methodology to distinguish intrahepatic saturated, mono‐ and polyunsaturated fatty acids in vivo and to assess the relationship between hepatic lipid composition and DNL.^10^ Participants were excluded if they had an active illness, participated in an exercise programme for more than 2 h per week, had significant weight change prior to enrolment, consumed more than two units of alcohol per day or smoked more than five cigarettes per day, used anti‐coagulants or other medication that interferes with hepatic lipid composition, or had high serum TG levels (>4 mmol/L). Participants were instructed to refrain from alcohol consumption or physical exercise for two days prior to the measurements and to consume a standardized high carbohydrate dinner the evening prior to the measurements. They visited the metabolic research ward after an overnight fast.^10^

Anthropometrics, measurements of serum lipids and glucose and quantification of IHL content (expressed as the ratio CH_2_/H_2_0) by ^1^H‐MRS were performed as previously described.^10^ Serum insulin was measured with an automated chemiluminescent immunometric assay on the Immulite XPi instrument (Siemens Healthcare Diagnostics).

DNL was quantified by oral ingestion of deuterated water (2.86 g/kg body weight; 70% ${\;}^{2}H_{2}O$, Cambridge Isotope laboratories) the evening prior to the measurement. DNL was quantified by the isotopic enrichment ratio of VLDL‐TG palmitate, measured with gas chromatography‐mass spectrometry (Agilent; Model 6890N/5975B).^13^

### 2.4 Statistical analyses

Continuous data are presented as mean ± standard deviation (SD) or as median (interquartile range) in case of non‐normal distribution. Categorical data are presented as frequencies. Non‐normally distributed variables were log‐transformed before further analyses. Multivariable regression analyses were performed to study the association between DNL and serum SHBG, adjusted for study population (Oxford or Maastricht), age, BMI and fasting insulin levels. Given the well‐known sex differences in SHBG levels, all primary analyses were stratified by sex. A potential interaction between sex and DNL on serum SHBG was formally tested by adding an interaction term (sex × DNL) to the regression model in the overall population, that is men and women combined.

All results were considered statistically significant at *p* < .05, except for interaction terms (*p* < .10). All statistical analyses were performed using IBM Statistical Package of Social Science (SPSS) version 25.0 for Windows (IBM Corp.).

## 3 RESULTS

### 3.1 Study population characteristics

**FIGURE 1:**
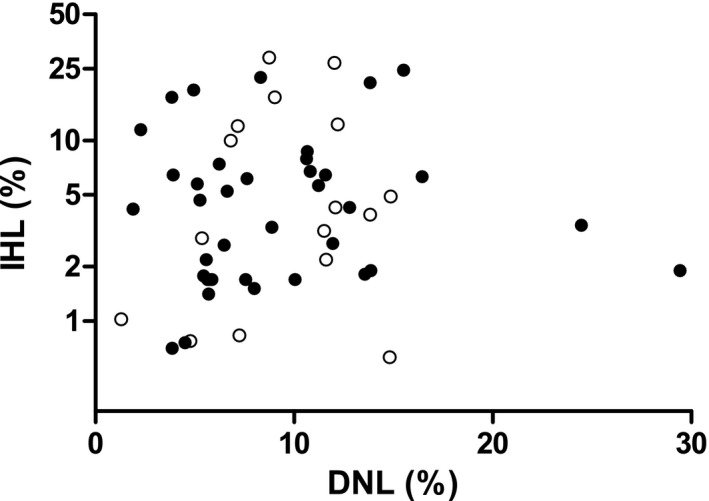
Association between de novo lipogenesis (DNL) and intrahepatic lipid (IHL) content stratified by study population, that is Oxford (closed circles) and Maastricht (open circles)

Three participants of the original Oxford (*n* = 41) and Maastricht (*n* = 17) cohorts were excluded from further analyses due to insufficient serum to determine SHBG (*n* = 1, Oxford) or serum TG >4 mmol/L (*n* = 1, Maastricht; *n* = 1, Oxford). The general characteristics of the Oxford and Maastricht study populations are presented in Table 1. Maastricht participants were older and more overweight compared with the Oxford cohort. BMI distribution in the combined cohort ranged from lean (BMI <25 kg/m^2^: 6/55 [11%]), overweight (BMI ≥25 and <30 kg/m^2^: 34/55 [62%]) to obese (BMI ≥30 kg/m^2^: 15/55 [27%]). Twenty‐two out of 55 (40%) individuals had an IHL content above the cut‐off value for hepatic steatosis (ie >5.56% IHL content).^7^ None of the female participants used oral contraceptives. Serum insulin levels were substantially higher in the Oxford cohort, which is most likely explained by a difference in assay. There was no statistically significant association between DNL and IHL contents (β: 0.008, 95% CI: −0.016; 0.032; adjusted for study population; Figure 1). Stratification by sex showed similar results in men (β: 0.000, 95% CI: −0.029; 0.029) and women (β: 0.014, 95% CI: −0.030; 0.059).

**TABLE 1:** Characteristics of Oxford and Maastricht study populations

	Oxford (*n* = 39)	Maastricht (*n* = 16)
Male/female (*n*)	28/11	6/10
Age (y)	44.2 ± 6.4	59.3 ± 7.0
BMI (kg/m^2^)	27.9 ± 2.9	29.6 ± 2.2
Glucose (mmol/L)	5.3 ± 0.5	5.5 ± 0.6
Insulin (pmol/L)	83.9 (60.3–99.2)	54.8 (32.3–85.0)
Total cholesterol (mmol/L)	5.4 ± 0.9	5.6 ± 1.1
LDL‐cholesterol (mmol/L)	3.9 ± 0.8	4.0 ± 1.1
HDL‐cholesterol (mmol/L)	1.1 ± 0.3	1.3 ± 0.4
Triglycerides (mmol/L)	1.8 (1.1–2.2)	1.6 (1.3–2.5)
Intrahepatic lipids (%)	4.1 (1.7–6.8)	4.1 (1.3–12.2)
De novo lipogenesis (%)	7.6 (5.3–11.6)	10.3 (6.9–12.2)
SHBG (nmol/L)	28.1 (22.8–36.1)	38.2 (31.6–57.3)
Use of oral contraceptives (*n*)	0	0

Note

Data are expressed as mean ± SD or as median (interquartile range)

Abbreviations: BMI, body mass index; SHBG, sex hormone binding globulin.

### 3.2 Relationship between DNL and serum SHBG levels

**FIGURE 2:**
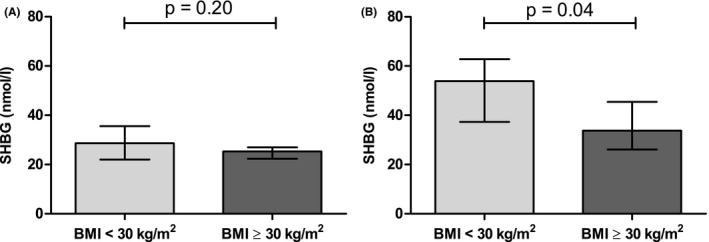
Sex hormone binding globulin (SHBG) levels in obese (body mass index [BMI] ≥30 kg/m^2^) and non‐obese (BMI <30 kg/m^2^) men (A) and women (B). Data are expressed as median with interquartile range. Differences between groups were analysed with linear regression analyses, adjusted for study population

In the combined cohort, serum SHBG levels were not significantly different between men with and without obesity, whereas SHBG levels were lower in women who were obese (β: −0.083, 95% CI: −0.212; 0.046; Figure 2A and β: −0.183, 95% CI: −0.361; −0.005; Figure 2B, respectively; adjusted for study population).

**FIGURE 3:**
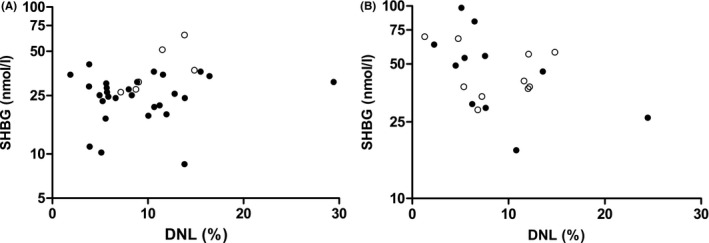
Associations between de novo lipogenesis (DNL) and sex hormone binding globulin (SHBG) in men (A) and women (B). Data are stratified by study cohort, that is Oxford cohort (closed circles) and Maastricht cohort (open circles)

In men, there was no statistically significant association between DNL and serum SHBG levels (β: 0.007, 95% CI: −0.005; 0.019; Table 2, Figure 3A). Similar trends were observed when the study populations, that is Oxford and Maastricht, were analysed separately (β: 0.003, 95% CI: −0.009; 0.015 and β: 0.038; 95% CI: −0.009; 0.085, respectively; Figure 3A). Further adjustment for study population, age, BMI and serum insulin did not alter the results (β: 0.002, 95% CI: −0.009; 0.014; Table 2). In women, there was a statistically significant, inverse association between DNL and serum SHBG (β: −0.015, 95% CI: −0.030; 0.000; Table 2, Figure 3B). The strength of association was statistically significantly different from men (*p* for interaction = .068). Similar trends were observed when the study populations were analysed separately (β: −0.019, 95% CI: −0.043; 0.004 and β: −0.006, 95% CI: −0.003; 0.019 for Oxford and Maastricht study population, respectively; Figure 3B). The strength of the association did not materially change after further adjustment for study population, age and BMI, although statistical significance was lost after further adjustment for serum insulin (β: −0.013, 95% CI: −0.028; 0.003; Table 2). Of note, serum insulin was not an independent determinant of serum SHBG in this fully adjusted model (*p* = .219).

**TABLE 2:** Association of de novo lipogenesis with (log) sex hormone binding globulin in men and women

Model, independent variables	Men (*n* = 34)		Women (*n* = 21)	
	β	95% CI	β	95% CI
Crude	0.007	−0.005; 0.019	−0.015	−0.030; 0.000
Model 1: study population (Oxford/Maastricht)	0.005	−0.006; 0.016	−0.015	−0.031; 0.000
Model 2: model 1 + age	0.001	−0.011; 0.012	−0.015	−0.031; 0.001
Model 3: model 2 + BMI	0.002	−0.010; 0.014	−0.018	−0.031;−0.006
Model 4: model 3 + serum insulin	0.002	−0.009; 0.014	−0.013	−0.028; 0.003

## 4 DISCUSSION

The aim of this study was to examine the relationship between DNL and serum SHBG in humans. We found an inverse association between DNL, measured with stable isotopes, and serum SHBG in women but not in men.

The current findings support and extend previous in vitro and animal studies, showing that DNL is involved in SHBG regulation. In vitro studies have demonstrated that monosaccharide‐induced DNL in HepG2 cells resulted in downregulation of hepatocyte nuclear factor‐4alpha (HNF‐4α) and, consequently, reduced expression of SHBG.^8^ Similar results were obtained after incubation with palmitate, a saturated fatty acid that is the end product of DNL.^8^ In the present study, we found that incorporation of deuterium into VLDL‐TG palmitate, a measure of DNL, was inversely associated with serum SHBG levels in women.

We observed a statistically significant interaction between sex and DNL on serum SHBG levels. The inverse relationship between DNL and serum SHBG was observed in women, but not in men. Strikingly, the inverse association between obesity and SHBG was also more pronounced in women. These sex differences may be accidental and, hence, deserve further replication. Alternatively, the difference between men and women may be the result of biological differences in transcriptional regulation of SHBG between men and women.^14^ It has been suggested that HNF‐4α, the oestrogen receptor alpha, and PPARG compete for binding to the SHBG promotor, with the former two stimulating and the latter inhibiting *SHBG* gene expression.^15^ The net effect of this competition on serum SHBG levels is difficult to predict and deserves further investigation.

Nevertheless, we postulate that the relationship between DNL and serum SHBG in women is of particular interest as it may provide a mechanistic link between obesity, more specifically hepatic fat accumulation and polycystic ovary syndrome (PCOS). Previous observational studies have shown that patients with PCOS have a high IHL content.^16^ A recent Mendelian randomization studies have inferred a causal relationship between low serum SHBG levels and PCOS risk.^17^ Of note, it is likely that factors other than DNL, such as tumour necrosis factor α and interleukin 1β, also contribute to the decreased serum SHBG levels in obesity and related disorders.^18^, ^19^

In this study, insulin did not appear to be a major contributor of serum SHBG levels. To date, a large body of literature has reported an inverse association between serum insulin and serum SHBG levels in humans.^20^ It is, however, virtually impossible to distinguish a potential direct effect of insulin on SHBG expression from confounding in an observational study design, particularly because insulin also affects DNL.^21^, ^22^ Although statistical significance was lost when insulin was added to the model as a potential confounder of the relationship between DNL and serum SHBG in women, the effect size for that relationship was hardly affected (the beta coefficient decreased from −0.018 to −0.013), which indicates that insulin is not a major contributor. A lack of statistical power, as a result of adjustment for multiple variables, is more likely. Indeed, serum insulin was not an independent determinant of serum SHBG in this cohort.

In the present study we did not observe an association between DNL and IHL content. IHL content is the net result of the influx of lipids—via DNL and FFA from adipose tissue—and the efflux of lipids—via beta‐oxidation and VLDL secretion.^7^ Each pathway is regulated by many genetic, environmental and hormonal factors.^7^ The original Oxford study showed that, as a result of differential partitioning of fatty acids in the liver, higher rates of DNL are not necessarily reflected by an increased IHL content.^9^ The authors speculated that this may be the result of preferential channelling of de novo synthesized fatty acids towards VLDL secretion rather than hepatic storage.^9^

This study has several strengths and limitations. First, by combining data from two study populations, that is Oxford and Maastricht, we were able to create a relatively large cohort to study the sex‐specific relationship between DNL, assessed with stable isotopes, and serum SHBG. Although differences between the cohorts may exist, regression analyses were adjusted for study population, which did not affect the strength of the association. In addition, stratified analyses in the Oxford and Maastricht cohort yielded similar trends. Second, although none of the included women used oral contraceptives, which are known to significantly affect SHBG levels,^23^ we did not have information on postmenopausal status or phase of menstrual cycle. Previous studies have shown that menopausal status does not seem to have an independent effect on DNL or SHBG levels.^24^, ^25^, ^26^ Furthermore, other studies have shown that DNL varies significantly throughout the menstrual cycle, while levels of SHBG remain constant.^27^, ^28^ Despite these scattering effects, a significant, inverse association was observed between DNL and SHBG in women.

In conclusion, in the present study we corroborate and extrapolate findings from previous in vitro and animal studies by showing that DNL is inversely associated with serum SHBG in women.

## AUTHOR CONTRIBUTIONS

PIHGS analysed the data and drafted the manuscript. MRT, JB, PS, PV, and VBSH contributed to the original Maastricht study, LH contributed to the original Oxford study. OV, IT, KMvdW, DMdG, PV, JAPB, MRT, JB, PS, JHWR, DC, CGS, CDAS, VBSH and LH provided critical feedback for the manuscript. MCGJB conceived the study design, supervised the analyses, and provided substantial revisions to the manuscript.

## CONFLICT OF INTEREST

The authors declare that there is no conflict of interest.

## Data Availability

The data sets generated during and/or analysed during the current study are not publicly available but are available from the corresponding author on reasonable request.
